# Acyclovir-Induced Nephrotoxicity and Neurotoxicity: A Report of Two Cases

**DOI:** 10.7759/cureus.52367

**Published:** 2024-01-16

**Authors:** Andrew Pei Wern Lim, Jaeeun Sung, Vivek Ramburuth, Samson O Oyibo

**Affiliations:** 1 General Internal Medicine, Peterborough City Hospital, Peterborough, GBR; 2 General Medicine, Peterborough City Hospital, Peterborough, GBR; 3 Diabetes and Endocrinology, Peterborough City Hospital, Peterborough, GBR

**Keywords:** acyclovir crystals, overweight, obese, adjusted body weight, actual body weight, ideal body weight, neurotoxicity, nephrotoxicity, intravenous acyclovir

## Abstract

Acyclovir is a widely used antiviral agent used to treat viral meningitis. Although well tolerated, on rare occasions, it can cause severe nephrotoxicity and neurotoxicity. It is recommended that the dose of intravenous acyclovir be calculated based on the ideal body weight for height rather than the actual weight in obese patients to avoid excessive dosage. We report two patients who developed severe acute kidney injury and neurological symptoms while on intravenous acyclovir therapy. The first patient was a 57-year-old obese woman known to have epilepsy who received a dose of intravenous acyclovir based on her actual weight of 80 kg and subsequently developed acyclovir-induced nephrotoxicity and increased seizure activity. The second patient was a 60-year-old, slightly overweight, man, who received a dose of intravenous acyclovir based on his actual weight of 80 kg and subsequently developed both acyclovir-induced nephrotoxicity and possible neurotoxicity. No other cause for the deterioration in renal function or neurological symptoms was identified, and there was rapid recovery within three days of stopping acyclovir therapy. This case report emphasizes the importance of monitoring renal function while patients are on intravenous acyclovir therapy and highlights the fact that even non-obese, overweight patients are at risk of toxicity when their actual body weight instead of their ideal body weight for height is used for intravenous acyclovir dose calculation.

## Introduction

Acyclovir is a common antiviral medication that is used to treat infections caused by herpes simplex and varicella viral infections, including meningitis. Acute kidney injury is a rare side effect of intravenous acyclovir use. The mechanism for renal toxicity is mainly through the formation of tubular crystal deposits, which causes an acute rise in creatinine, commonly in the first 24-48 hours of administration [1-3]. Risk factors for acyclovir-induced nephrotoxicity include intravascular volume depletion, rapid intravenous administration, hypertension, underlying renal disease, and obesity [3,4]. Renal failure can be reversed by discontinuing the use of acyclovir and prompting treatment with intravenous hydration and urine alkalization. Dialysis is rarely required [5].

The dose of acyclovir is calculated based on body weight and is usually at a dose of 10 mg/kg body weight in adults [6]. The manufacturer’s labeling recommends using ideal weight for height rather than actual weight in obese patients with a BMI greater than 29.9 kg/m^2^ to avoid excessive dosage [7]. The use of actual body weight in obese patients results in two-fold higher serum concentrations compared with non-obese patients [7]. We describe two patients who developed acute nephrotoxicity and possible neurotoxicity while on intravenous acyclovir therapy. The first case is a 57-year-old woman who was obese and was erroneously given intravenous acyclovir based on her actual body weight. The second case is a 60-year-old man who was non-obese but overweight and given intravenous acyclovir based on his actual body weight.

## Case presentation

Case 1

A 57-year-old woman presented with generalized tonic-clonic seizures, fever, and an altered mental state. She had a past medical history of both epileptic seizures and non-epileptic seizures, well controlled on both lacosamide and topiramate. On examination, she was agitated with a temperature of 38.5°C, a heart rate of 68 beats per minute, a respiratory rate of 20 breaths per minute, and a blood pressure of 136/71 mmHg. Examination of her chest and abdomen revealed no abnormalities. The central nervous system examination did not demonstrate any clinical signs of meningitis. She weighed 80 kg (height of 1.57 m) with a BMI of 32.5 kg/m^2^.

Initial blood investigations demonstrated leukocytosis (neutrophilia), a slightly elevated C-reactive protein level, and normal liver and renal function (Table 1). Blood and urine cultures were negative. A computed tomography scan of her brain was normal.

**Table 1:** Initial investigations ALP: alkaline phosphatase; ALT: alanine transferase; eGFR: estimated glomerular filtration rate

Blood test	Result			Reference range
Sodium (mmol/L)	135			132-145
Potassium (mmol/L)	3.5			3.4-5.1
Chloride (mmol/L)	105			97-110
Creatinine (μmol/L)	55			45-84
Urea (mmol/L)		4.5	2.5-7.8	
Thyroid-stimulating hormone (mU/L)		2.2	0.3-4.2	
C-reactive protein (mg/L)		15	<5	
eGFR (ml/min)		>90	>60	
Total protein (g/L)		78	60-80	
Albumin (g/L)		46	35-50	
ALT (U/L)		30	<33	
ALP (U/L)		81	30-130	
Bilirubin (μmol/L)		5	0-21	
Hemoglobin (g/L)		157	115-165	
White blood cell count (10^9^/L)		19.7	4.0-11.0	
Platelet count (10^9^/L)		258	150-400	
Prothrombin time ratio		1.05	0.8-1.25	

Meningitis was suspected, and she was started on intravenous ceftriaxone 2 grams twice a day and intravenous acyclovir 800 mg (10 mg/kg) three times a day. The acyclovir dose was calculated using her actual body weight. Subsequent CSF examination was negative for bacteria; ceftriaxone was therefore stopped, but acyclovir was continued, with the intention to stop if the virology was negative.

**Figure 1:**
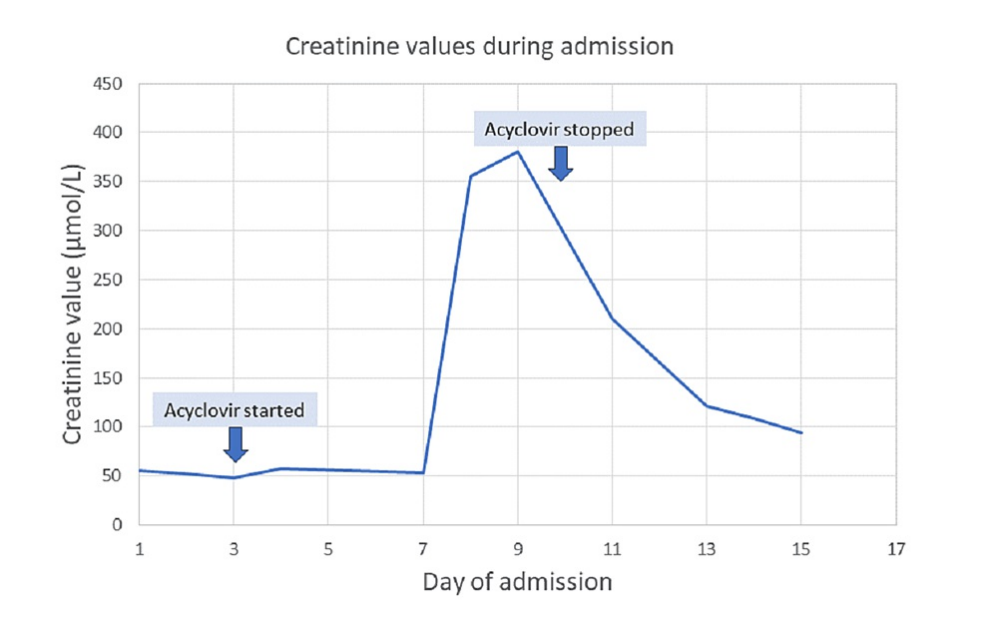
Renal function throughout the hospital stay Showing when intravenous acyclovir was started and stopped

On the eighth day of admission (five days after starting acyclovir), a blood test was performed because of further seizure activity. This revealed an acute kidney injury with a creatinine value of 355 µmol/L and an estimated glomerular filtration rate (eGFR) of 12 ml/min. The eGFR value fell to a nadir of 11 ml/min with a peak creatinine value of 381 µmol/L (Figure 1). Other blood results remained stable. An autoimmune kidney screen was negative. Paracetamol and salicylate levels were normal. Urinalysis demonstrated red blood cells in the urine but no nitrates, proteins, or leukocytes. A renal ultrasound examination demonstrated normal kidneys.

This patient had developed acyclovir-induced nephrotoxicity. The increased seizure activity could have been related to acyclovir toxicity or impaired renal function. The patient was reviewed by the nephrologist and commenced on an intravenous fluid infusion. The acyclovir dose was continued at a reduced dose of 550 mg three times a day in light of the kidney injury. It was not stopped for fear of inadequately treated viral meningitis. The acyclovir was later stopped on the 10th day of admission after the CSF virology was reported to be negative. Three days later, the kidney function improved (eGFR of 43 ml/min; creatinine of 94 µmol/L). Subsequent renal function tests demonstrated a complete recovery.

Case 2

A 60-year-old man presented with a five-day history of headache, photophobia, vomiting, and a rash over both arms. He had a flu-like illness three weeks prior to this presentation. He had no other symptoms on the systemic review. His past medical history included gout and psoriasis, for which he was on allopurinol 100 mg daily. He was allergic to diclofenac. He was otherwise fit and well; he did not smoke but drank alcohol occasionally. On examination, he was clinically stable: a temperature of 36.8°C, a heart rate of 115 beats per minute, a respiratory rate of 17 breaths per minute, and a blood pressure of 161/101 mmHg. Examination of his chest and abdomen revealed no abnormalities. The central nervous system examination did not demonstrate any clinical signs of meningitis. He weighed 80 kg (height of 1.73 m) with a BMI of 26.7 kg/m^2^.

Initial investigations revealed mild leukocytosis (neutrophilia) with normal C-reactive protein levels. Coagulation screen, liver function, and renal function tests were normal (Table 2). Hepatitis serology was negative. Blood and urine cultures were negative. A computed tomography scan of his brain was normal.

**Table 2:** Initial investigations ALP: alkaline phosphatase; ALT: alanine transferase; eGFR: estimated glomerular filtration rate

Blood test	Result		Reference range
Sodium (mmol/L)	138		132-145
Potassium (mmol/L)	4.0		3.4-5.1
Chloride (mmol/L)	102		97-110
Creatinine (μmol/L)	70		45-84
Urea (mmol/L)		3.8	2.5-7.8
C-reactive protein (mg/L)		<1	<5
eGFR (ml/min)		>90	>60
Total protein (g/L)		61	60-80
Albumin (g/L)		36	35-50
ALT (U/L)		21	<33
ALP (U/L)		67	30-130
Bilirubin (μmol/L)		16	0-21
Hemoglobin (g/L)		162	115-165
White blood cell count (10^9^/L)		11.7	4.0-11.0
Platelet count (10^9^/L)		210	150-400
Prothrombin time ratio		0.93	0.8-1.25

After having a lumbar puncture performed for CSF examination, he was commenced on intravenous ceftriaxone 2 grams twice a day and acyclovir 800 mg (10 mg/kg) three times a day to cover for bacterial and viral meningitis. The acyclovir dose was calculated using his actual body weight.

**Figure 2:**
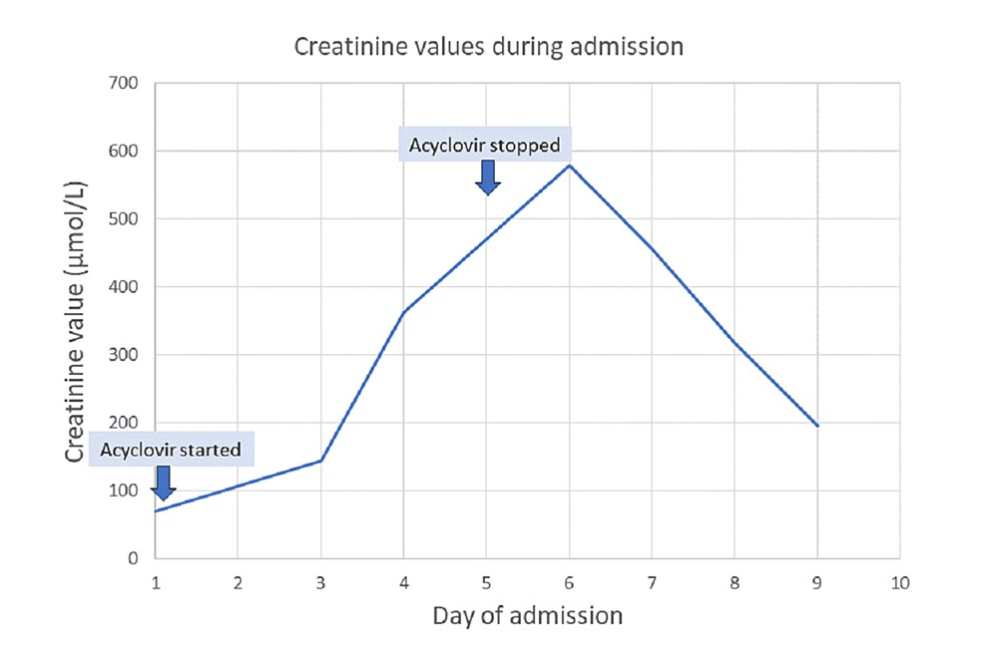
Renal function throughout the hospital stay Showing when intravenous acyclovir was started and stopped

Two days after commencing acyclovir and ceftriaxone, the patient exhibited signs of confusion, agitation, and hallucinations. A repeat blood test revealed acute kidney injury with a creatinine value of 144 µmol/L and an eGFR of 45 ml/min. By the next day, the eGFR value fell to 15 ml/min and then to a nadir of 8 ml/min with a peak creatinine value of 597 µmol/L (Figure 2). Other blood results remained stable. A CSF examination did not demonstrate any bacterial or viral infection. An autoimmune kidney screen was negative. Urinalysis demonstrated red blood cells in the urine; the urine was negative for nitrates, proteins, and leukocytes. A renal ultrasound examination demonstrated normal kidneys.

This patient had developed acyclovir-induced nephrotoxicity plus probable neurotoxicity. Initially, it was thought that the neurotoxic symptoms were due to viral encephalitis. The patient was reviewed by the neurologist and nephrologist and commenced on an intravenous fluid infusion. The acyclovir was stopped while he completed the course of ceftriaxone. Three days later, the patient was much better. By the end of the second week, his renal function was back to normal.

## Discussion

We have presented two cases of severe acyclovir-induced nephrotoxicity and possible neurotoxicity. Both patients had a BMI above the normal range of 18-24.9 kg/m^2^. In both cases, the actual body weight was used instead of the ideal body weight for height for calculating the acyclovir dose. The patient in Case 1 was obese with an actual body weight of 80 kg and had an acyclovir dose of 800 mg three times a day. Her ideal body weight for height was 49.7 kg using Devine’s formula [8]. If her ideal body weight was used, she would have received a much lower dose (500 mg three times a day). The patient in Case 2 was overweight (non-obese) with an actual body weight of 80 kg and had an acyclovir dose of 800 mg three times a day. His ideal body weight for height was 68.7 kg. If his ideal body weight was used, he would have received a much lower dose (690 mg three times a day). Therefore, the patient in Case 1 received an acyclovir dose 60% more than if the ideal body weight for height was used, and the patient in Case 2 received an acyclovir dose 16% more than if the ideal body weight for height was used.

Mechanisms for acyclovir-induced nephrotoxicity have been postulated, such as drug-induced acute tubular necrosis or direct injury from the aldehyde metabolite of acyclovir. However, the theory most extensively studied is crystal nephropathy [9-11]. While 5-15% of acyclovir is metabolized in the liver, the drug is mostly excreted via the kidneys in its unmetabolized form. Acyclovir is relatively insoluble in urine, especially in the distal tubular lumen, where it forms crystal deposits [9-11]. These crystals, which are needle-shaped with birefringence when visualized under polarized light urine microscopy, can cause nephron obstruction, interstitial congestion, and hemorrhage in the tubular parenchyma, resulting in kidney injury [9-11], Acyclovir-induced nephropathy is more common with intravenous administration due to its higher bioavailability, but there are rare reports of toxicity occurring after oral administration [12,13].

Although a rare complication, there have been several case reports describing the occurrence of acute kidney injury after administration of acyclovir, with some cases requiring hemodialysis [14]. A single-center retrospective cohort study in 2018 revealed that the median time to develop acyclovir-induced nephrotoxicity was two days, with a range of 1-14 days [15]. In the same cohort study, it was noted that in the patients who developed nephrotoxicity, most of them developed deranged renal function within a week of starting acyclovir therapy [15]. Our first patient (Case 1) demonstrated nephrotoxicity on the fifth day of starting acyclovir. Our case study thus reinforces this observation that nephrotoxicity can develop well beyond two to three days after the first dose of intravenous acyclovir.

Neurotoxicity is a rarer complication of acyclovir administration, but a few papers have described it manifesting as agitation, tremors, myoclonus, confusion, hallucinations, or seizures [16,17]. It must be noted that these symptoms may be misinterpreted as symptoms of viral encephalitis. While our first patient (Case 1) did have an increased frequency of seizures, it is difficult to tell whether this was directly caused by acyclovir itself or because of the rapid rise in creatinine. This was further complicated by her background history of non-epileptic seizures and epileptic seizures. Our second patient (Case 2), however, had severe symptoms of neurotoxicity initially thought to be related to viral meningitis, which improved soon after stopping acyclovir.

The dechallenge test was positive for both cases (i.e., improvement of serum creatinine and neurological symptoms after stopping the acyclovir). However, other possible causes for the neurological symptoms could not be completely ruled out. Hence, as per the World Health Organization-Uppsala Monitoring Center (WHO-UMC) system for standardized case causality assessment, the causal relationship between the administration of acyclovir and the onset of nephrotoxicity in this case would be regarded as probable or likely, while the causal relationship between the administration of acyclovir and the onset of neurotoxicity in this case would be regarded as possible [18].

There is limited guidance and still debate about the appropriate dosing strategy for intravenous acyclovir in obese patients [7,19]. Weight-based dosing in obese patients is complicated by the choice of using ideal body weight for height, total (actual) body weight, and adjusted body weight [7,19]. Use of the wrong weight can result in overdosing or underdosing. There is also concern that the use of ideal body weight for height can lead to subtherapeutic concentrations in obese patients when compared to non-obese patients [19]. The actual body weight was erroneously used for the patient in Case 1; the ideal weight for height should have been used for acyclovir dosing. However, the patient in Case 2 was non-obese, just about in the overweight category where actual body weight can still be used for acyclovir dosing. However, this patient still developed both nephrotoxicity and neurotoxicity. This interesting occurrence brings forth the question of whether the ideal body weight for height should be used for acyclovir dose calculation in overweight (BMI: 25-29.9 kg/m^2^) patients as well.

## Conclusions

Acyclovir is a commonly used drug and, although well tolerated, can cause severe nephrotoxicity and neurotoxicity. This case report highlights that nephrotoxicity can occur well after the usual median time frame of two days. Appropriate dosing and regular monitoring of renal function are essential for patients while on acyclovir therapy.

Additionally, there is a need for further clarification regarding the use of actual body weight versus ideal body weight for height or adjusted body weight for intravenous acyclovir dosing in non-obese, overweight patients.
